# Synergistic approach to polycycles through Suzuki–Miyaura cross coupling and metathesis as key steps

**DOI:** 10.3762/bjoc.14.223

**Published:** 2018-09-21

**Authors:** Sambasivarao Kotha, Milind Meshram, Chandravathi Chakkapalli

**Affiliations:** 1 Department of Chemistry, Indian Institute of Technology Bombay, Powai, Mumbai-400 076, India, Fax: +91(22)-2576 7152

**Keywords:** Claisen rearrangement, Diels–Alder reaction, metathesis, polycycles, Suzuki–Miyaura cross coupling

## Abstract

This account provides an overview of recent work, including our own contribution dealing with Suzuki–Miyaura cross coupling in combination with metathesis (or vice-versa). Several cyclophanes, polycycles, macrocycles, spirocycles, stilbenes, biaryls, and heterocycles have been synthesized by employing a combination of Suzuki cross-coupling and metathesis. Various popular reactions such as Diels–Alder reaction, Claisen rearrangement, cross-metathesis, and cross-enyne metathesis are used. The synergistic combination of these powerful reactions is found to be useful for the construction of complex targets and fulfill synthetic brevity.

## Introduction

**Figure 1:**
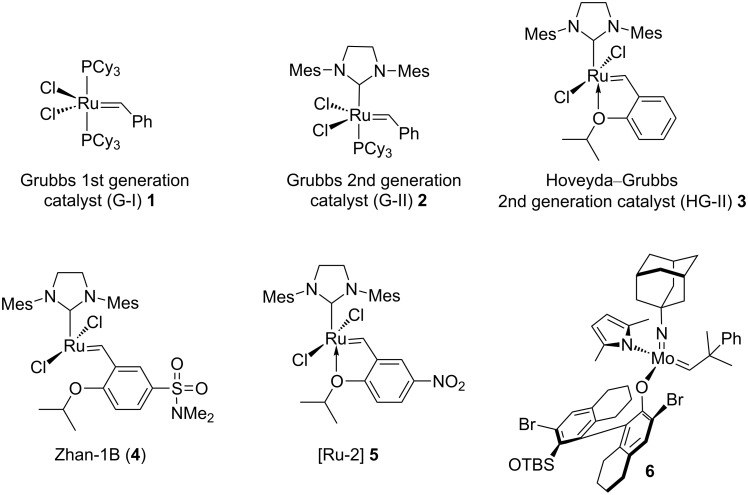
Various catalysts used for metathesis reactions.

Transition-metal catalysts are used in metathesis and cross-coupling reactions. Such advances have opened the door for efficient construction of C–C bonds in organic synthesis. These catalysts tolerate diverse functional groups and the reaction occurs under mild reaction conditions. Among different metathetic processes, ring-closing metathesis (RCM) [1–6] is of a greater interest than cross-metathesis (CM). It is a widely used protocol for the synthesis of unsaturated cyclic systems [7]. Palladium-catalyzed Suzuki–Miyaura (SM) cross-coupling reaction is also considered as one of the most versatile methods for C–C bond formation [8–12]. Application of a wide range of organometallic reagents (e.g., organoboron reagents) are possible due to their commercial availability. Owing to the mild reaction conditions and ease of handling of organoboron reagents [13–17] have propelled the growth of the SM cross coupling. A synergistic combination of these two elegant methods (i.e., SM coupling and metathesis) [18] was found to increase the synthetic efficiency of complex targets (e.g., macrocycles [19–22], oligomers [23–24], polycyclic ethers [25], heterocycles [26], nonbenzenoid aromatics [27], and spirocycles [28–29]) by decreasing the number of steps. Different metathesis catalysts used in this study are shown in Figure 1.

## Review

### Annulation

**Scheme 1:**
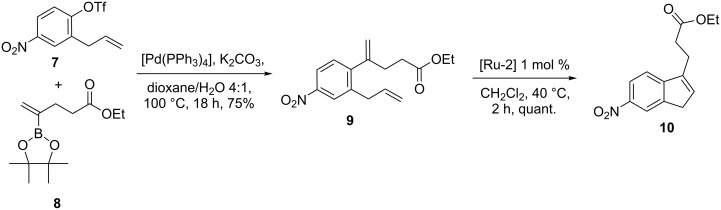
SM coupling and RCM protocol to substituted indene derivative **10**.

Grela and co-workers [30] demonstrated a useful protocol to build indene derivatives by employing SM coupling and RCM in sequence. To this end, the SM coupling of triflate **7** was accomplished by using pinacol boronic ester **8** in the presence of a palladium catalyst to give the cross-coupling product **9** (75%). Later on, exposure of the diolefinic precursor **9** to [Ru-2] catalyst **5** gave the ring-closure product **10** in quantitative yield (Scheme 1).

**Scheme 2:**
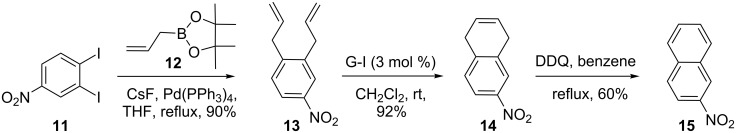
Synthesis of polycycles via SM and RCM approach.

A sequential usage of SM cross coupling and RCM was responsible to construct various naphthalene derivatives such as **15** [31]. The SM coupling product 3,4-diallylbenzene derivative **13** (90%) was obtained from diiodobenzene **11** using allylboronate ester **12** via a SM-type allylation sequence [32]. Next, compound **13** was exposed to Grubbs 1st generation (G-I) catalyst **1** to effect the ring-closure to produce tetrahydronaphthalene derivative **14** (92%). Subsequently, aromatization of compound **14** was accomplished with 2,3-dichloro-5,6-dicyano-1,4-benzoquinone (DDQ) to generate nitronaphthalene **15** (60%, Scheme 2).

**Figure 2:**
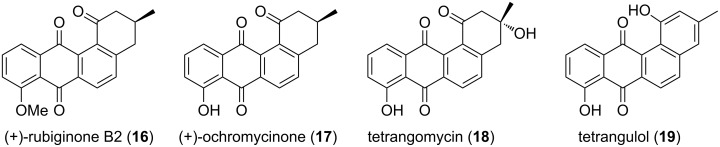
Various angucyclines.

**Scheme 3:**
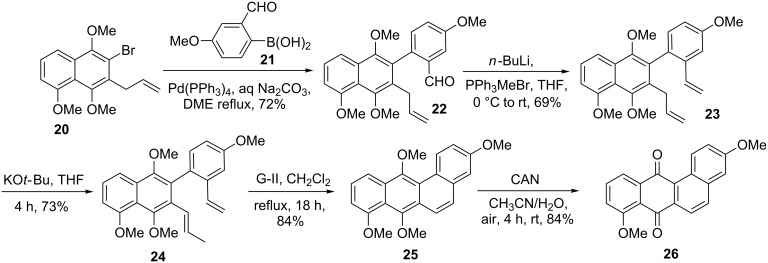
SM coupling and RCM protocol to the benz[*a*]anthracene skeleton **26**.

Due to their useful biological activity and intricate structural features of angucyclines such as **16–19** (Figure 2), several approaches have been reported for their assembly. In this context, de Koning and co-workers [33] demonstrated an efficient route for the construction of the benz[*a*]anthracene structural unit by employing SM cross coupling followed by RCM sequence. Treatment of the bromonaphthalene derivative **20** with (2-formyl-4-methoxyphenyl)boronic acid (**21**) in the presence of a palladium catalyst generated the cross-coupling product **22** (72%). Next, aldehyde **22** was subjected to Wittig olefination to provide the corresponding alkene **23** (69%), which on subsequent treatment with KO*t*-Bu in THF gave the isomerized product **24** (73%). Later, RCM of isomerized olefin **24** with the help of G-II catalyst offered the ring-closure product **25** (84%). Finally, CAN oxidation gave the desired tetracyclic compound **26** in 84% yield (Scheme 3).

### Spirocycles

**Scheme 4:**
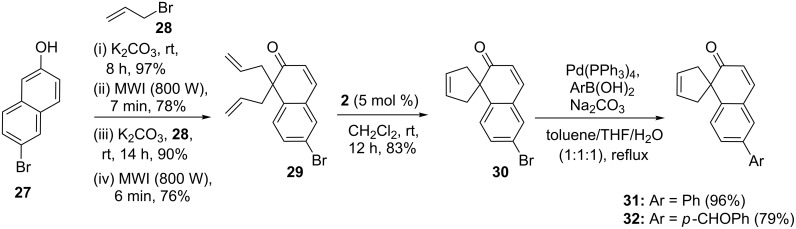
Synthesis of substituted spirocycles via RCM and SM sequence.

In another event, an efficient approach to spirocyclopentane derivatives has been described, where the combination of RCM and SM coupling was employed [34]. In this respect, the key building block **29** was derived by employing a sequential *O*-allylation and CR, then again *O*-allylation, and CR [35] starting with a commercially available 6-bromo-2-naphthol (**27**). Subsequently, the diallyl derivative **29** was exposed to G-II catalyst **2** to deliver a ring-closure product **30** (83%). Finally, the spiro compound **30** was subjected to the SM coupling using two different boronic acids to produce the aryl substituted spiro compounds such as **31** (96%) and **32** (79%) (Scheme 4).

**Scheme 5:**
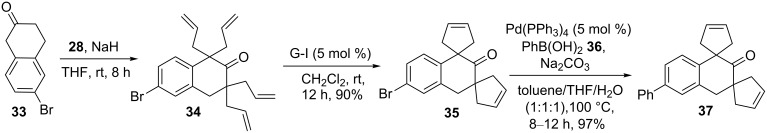
Synthesis of highly functionalized bis-spirocyclic derivative **37**.

Along similar lines, we have also demonstrated the synthesis of bis-spirocycles such as **37** by adopting a double RCM sequence followed by SM coupling [36]. The key precursor **34** was assembled from a commercially available tetralone **33** via tetraallylation sequence. Then, tetraallyl derivative **34** was subjected to RCM with the aid of the G-I catalyst **1** to furnish the bis-spirocyclic compound **35** (90%). Next, the cyclized product **35** was subjected to SM coupling using phenylboronic acid (**36**) to afford the cross-coupling product **37** (97%, Scheme 5).

**Scheme 6:**
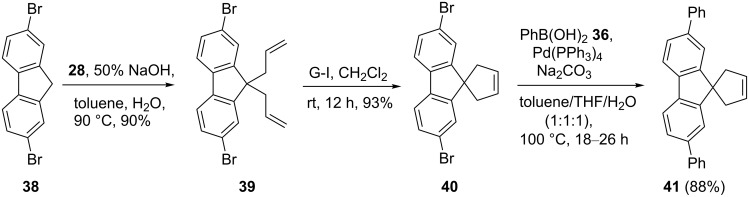
Synthesis of spirofluorene derivatives via RCM and SM coupling sequence.

In another instance, a simple synthetic approach to spiro-fluorene derivative **41** was described involving a serial usage of RCM and SM coupling [37]. To this end, bromofluorene **38** was reacted with allyl bromide (**28**) in the presence of 50% NaOH to deliver the expected 9,9′-diallylfluorene derivative **39** (90%). Next, diallyl compound **39** was subjected to RCM with the aid of the G-I catalyst **1** to furnish a ring-closure product, spirofluorene derivative **40** (93%). Later, the dibromide **40** was subjected to SM coupling in the presence of phenylboronic acid (**36**) to generate the new spirofluorene **41** (88%, Scheme 6).

**Scheme 7:**
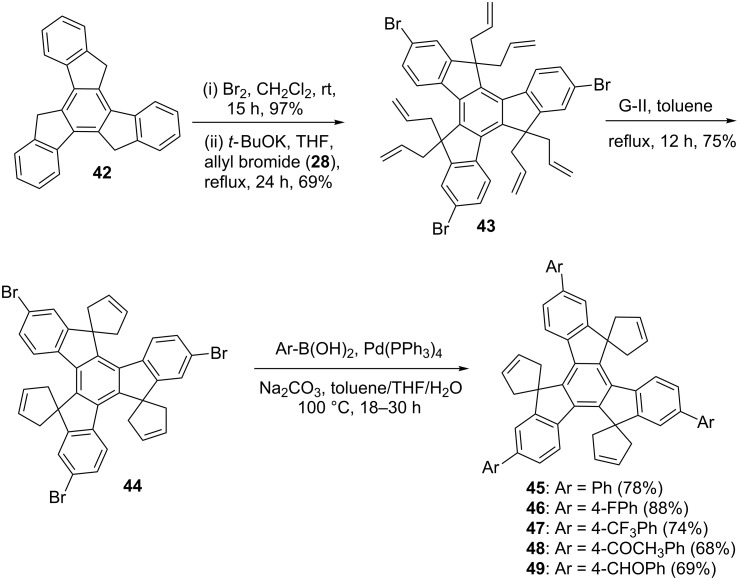
Synthesis of truxene derivatives via RCM and SM coupling.

Interestingly, highly substituted truxene derivatives **45–49** were also synthesized by applying the RCM and SM coupling protocol (Scheme 7).

### Heterocycles

**Scheme 8:**
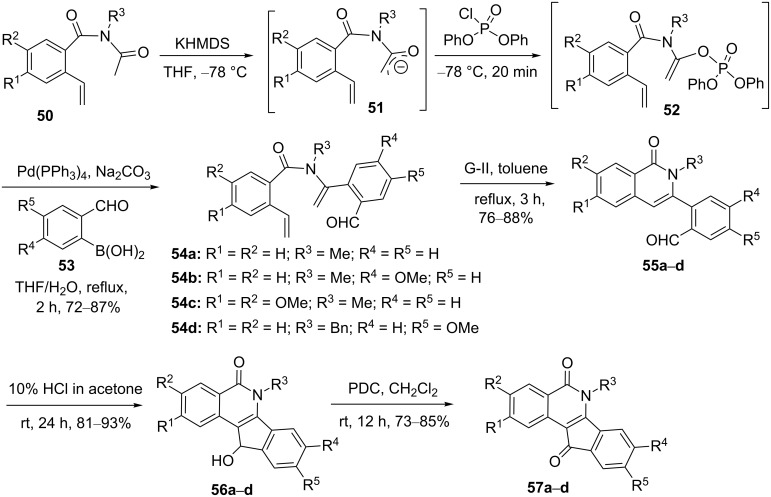
Synthesis of substituted isoquinoline derivative via SM and RCM protocol.

Couture and co-workers [38] demonstrated an elegant approach to highly substituted isoquinolones (e.g., **57a–d**, Scheme 8) by employing a SM coupling followed by RCM. To this end, they started with *o*-vinylbenzoic acid and it was transformed to the benzamide derivatives **50** by employing a four-step synthetic sequence. Later, compound **50** was treated with KHMDS in THF at −78 °C to produce enolate **51**. Further, it was reacted with diphenyl chlorophosphate to generate vinyl phosphate **52**, which was subjected to SM coupling in the presence of different 2-formylboronic acids **53** with the aid of the Pd(PPh_3_)_4_ catalyst to provide the respective coupling products **54a–d** (72–87%). Next, exposure of the diolefins **54a–d** to G-II catalyst **2** delivered ring-closure products, isoquinolones **55a–d** (76–88%). Finally, the cyclized products **55a–d** were converted into the corresponding indeno[1,2-*c*]isoquinolin-5,11-diones **57a–d** (73–85%) through cyclization with the aid of HCl followed by pyridinium dichromate (PDC) oxidation (Scheme 8).

**Scheme 9:**
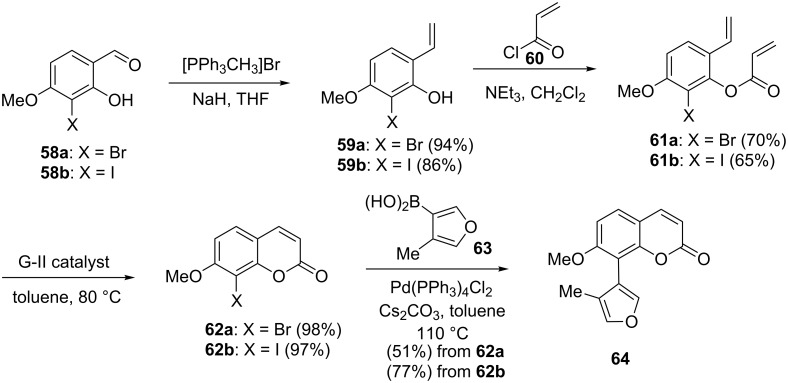
Synthesis to 8-aryl substituted coumarin **64** via RCM and SM sequence.

Schmidt and co-workers [39] described an efficient route involving RCM and SM coupling towards the synthesis of 8-aryl-substituted coumarin **64**, a natural product isolated from the plant *Galipea panamensis*. To this end, aldehydes **58a**,**b** were subjected to a Wittig olefination followed by condensation with acryloyl chloride (**60**) to generate the corresponding diolefinic substrates such as **61a** (70%) and **61b** (65%). Later, these diolefins **61a**,**b** were subjected to RCM with the aid of G-II catalyst **2** to furnish the respective ring-closure products **62a** (98%) and **62b** (97%). Finally, SM coupling of 8-halo-7-methoxycoumarins **62a**,**b** with (4-methylfuran-3-yl)boronic acid (**63**) delivered the cross-coupling product **64** (Scheme 9).

**Scheme 10:**
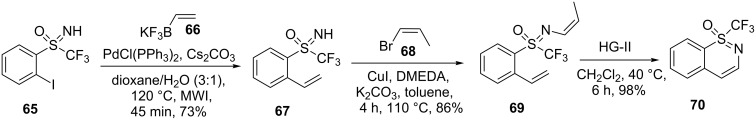
Synthesis of cyclic sulfoximine **70** via SM and RCM as key steps.

In another event, Magnier and co-workers [40] described a simple synthetic route to sulfoximines by adopting SM coupling and RCM as key steps. In this respect, SM coupling of sulfoximine **65** with potassium vinyltrifluoroborate (**66**) in the presence of a palladium catalyst produced vinyl sulfoximine derivative **67** (73%). Next, *N*-alkenylation of sulfoximine **67** was accomplished with *Z*-vinyl bromide (**68**) to generate diolefinic substrate **69** (86%). Finally, diolefin **69** was exposed to Hoveyda–Grubbs 2nd generation catalyst (HG-II) **3** to deliver the cyclic sulfoximine **70** in 98% yield (Scheme 10).

**Scheme 11:**
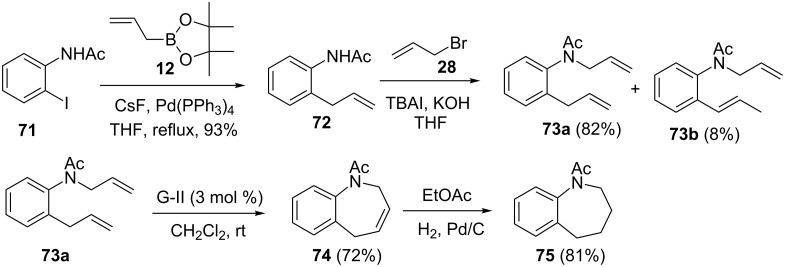
Synthesis of 1-benzazepine derivative **75** via SM and RCM as key steps.

Additionally, we also demonstrated a sequential usage of SM coupling and the RCM protocol to construct 1-benzazepine derivative **75** [41]. To this end, iodoacetanilide **71** was subjected to SM coupling in the presence of allyboronate ester **12** to give *ortho*-allylacetanilide (**72**), which was further modified by *N*-allylation with allyl bromide (**28**) to offer a mixture of diallyl compound **73a** (82%) and isomerized product **73b** (8%). Next, exposure of the diallyl derivative **73a** to G-II catalyst **2** yielded the cyclized product **74** (72%). Eventually, hydrogenation of the RCM product **74** was achieved with H_2_, Pd/C conditions to give the saturated 2,3,4,5-tetrahydro-1-benzazepine **75** in 81% yield (Scheme 11).

**Scheme 12:**
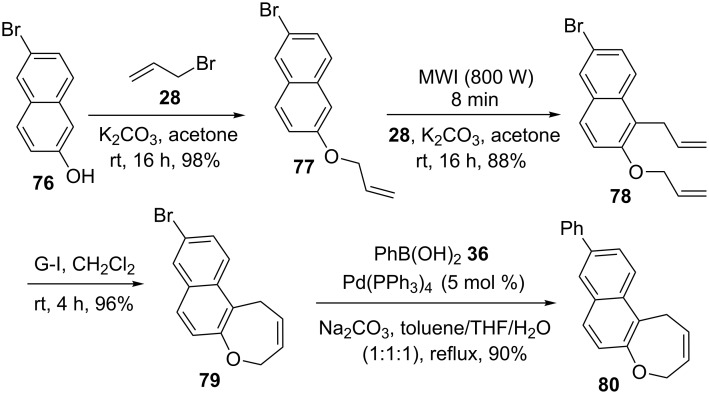
Synthesis of naphthoxepine derivative **79** via RCM followed by SM coupling.

Naphthoxepine derivatives play an important role as cosmetics and as pharmaceutical ingredients. Therefore, we conceived a simple approach, where the SM coupling and RCM were employed as critical steps [42–43]. Our journey begin with *O*-allylation of β-naphthol **76** by using allyl bromide (**28**) to give *O*-allyl derivative **77**. Then, Claisen rearrangement (CR) of **77** under microwave irradiation (MWI) conditions on a silica gel support followed by *O*-allylation of the resulting CR product furnished diallyl compound **78**. Treatment of diallyl compound **78** with G-I catalyst **1** delivered the expected naphthoxepine derivative **79** (96%). Next, Suzuki coupling of **79** with diverse arylboronic acids (e.g., phenylboronic acid (**36**)) gave a highly substituted naphthoxepine derivative **80** (90%) (Scheme 12).

### Stilbene derivatives

**Scheme 13:**
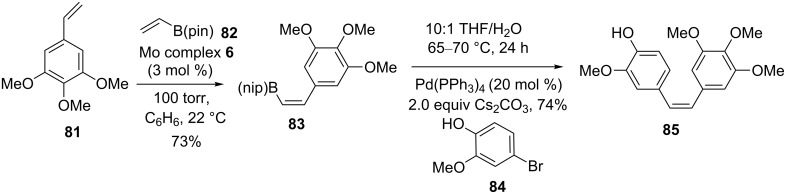
Sequential CM and SM coupling approach to *Z*-stilbene derivative **85**.

Hoveyda and co-workers [44] reported the synthesis of *Z*-(pinacolato)allylboron and *Z*-(pinacolato)alkenylboron derivatives via CM by using Mo complex **6**. In this regard, they assembled stilbene derivative **85** as an antitumor agent by a two-step strategy that involve catalytic CM and SM coupling. To this end, the *Z*-selective CM of a styrene derivative (e.g., **81**) with vinyl-B(pin) **82** was realized in the presence of Mo complex **6** to provide a highly substituted vinyl-B(pin) **83** (73%) with excellent selectivity (96:4 *Z*:*E*). Further, vinylboron compound **83** was subjected to SM coupling with a suitable partner (e.g., **84**) to afford the stilbene derivative **85** (96:4 *Z*:*E*) in 74% yield (Scheme 13).

**Scheme 14:**
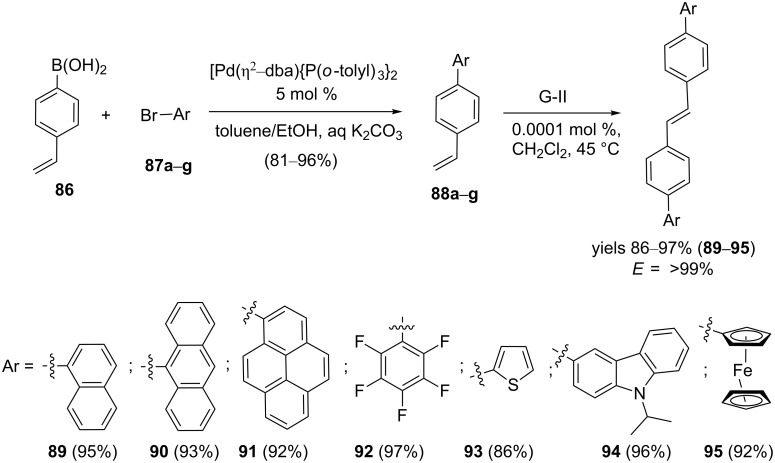
Synthesis of substituted *trans*-stilbene derivatives via SM coupling and RCM.

Majchrzak and co-workers [45] demonstrated a synergistic approach involving SM cross coupling and CM to synthesize various substituted *trans*-stilbene derivatives **89–95** stereoselectively. In this context, 4-vinylphenylboronic acid (**86**) was subjected to SM coupling using diverse bromoarenes **87a–g** in the presence of [Pd(η^2^-dba){P(*o*-tolyl)_3_}_2_] catalyst to obtain the cross-coupling products **88a–g** (81–96%). Finally, exposure of olefins **88a–g** to G-II catalyst **2** in CH_2_Cl_2_ led to the formation of the respective *trans*-stilbene derivatives **89–95** in high yields (Scheme 14). It is worth mentioning that the loading of only 0.0001 mol % catalyst can effect a CM in an efficient manner.

### Biaryl derivatives

**Scheme 15:**
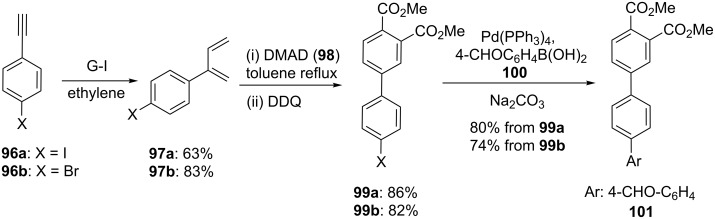
Synthesis of biaryl derivatives via sequential EM, DA followed by SM coupling.

In view of the interesting properties of biaryl derivatives, we have identified a three-step sequence, which involve cross-enyne metathesis (CEM), DA reaction followed by SM coupling [46]. To this end, acetylene derivatives **96a**,**b** were subjected to CEM with G-I catalyst **1** under ethylene, which resulted in the formation of the dienes **97a** (63%) and **97b** (83%, Scheme 15). Further, treatment of dienes **97a**,**b** with dimethyl acetylenedicarboxylate (DMAD, **98**) separately delivered the corresponding cycloadducts. Subsequently, aromatization was achieved by using DDQ to give biaryl products **99a**,**b**. Further, aryl halides **99a**,**b** were subjected to SM coupling by employing various boronic acids (e.g., 4-formylphenylboronic acid (**100**) to produce biaryl derivative **101** (80% from **99a** and 74% from **99b**).

**Scheme 16:**
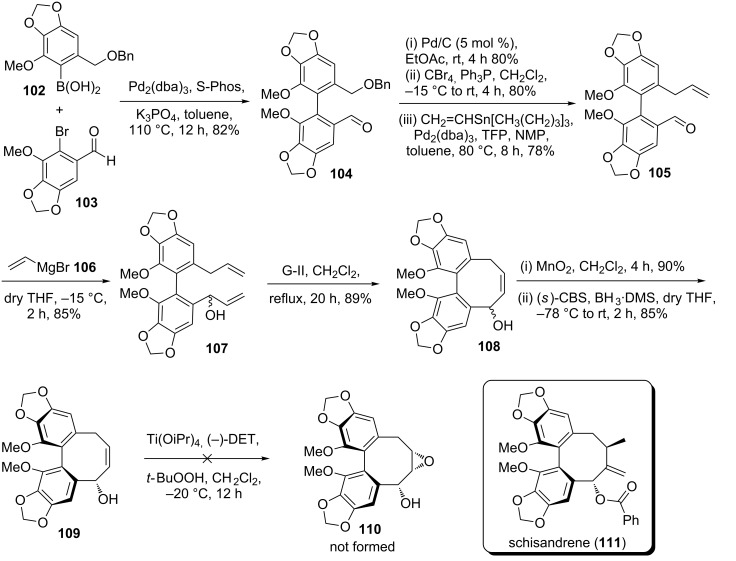
Synthesis of the dibenzocyclooctadiene core of schisandrene.

Very recently, Suresh Babu and co-workers [47] demonstrated a new route to construct the dibenzocyclooctadiene lignan core of the natural product schisandrene via SM coupling and RCM as key steps. In this context, the SM reaction of boronic acid **102** with bromoaldehyde **103** in the presence of Pd_2_(dba)_3_ and the S-Phos ligand provided the cross-coupling product **104** (82%). Later, it was transformed into the allyl substrate **105** by following a three-step sequence. Afterwards, the aldehyde **105** was treated with vinylmagnesium bromide (**106**) to furnish diallyl derivative **107** (85%). Next, diolefinic substrate **107** was exposed to G-II catalyst **2** to furnish the ring-closure product **108** (89%). Then, MnO_2_ oxidation of compound **108** offered the keto derivative in 90% yield. Corey–Bakshi–Shibata (CBS) reduction of the resulting keto derivative produced the hydroxy compound **109** (85%, ee 98%). Eventually, hydroxy olefin **109** was subjected to Sharpless asymmetric epoxidation to generate the corresponding epoxide **110**. Unfortunately, generation of epoxide was not realized (Scheme 16).

### Macrocycles

**Scheme 17:**
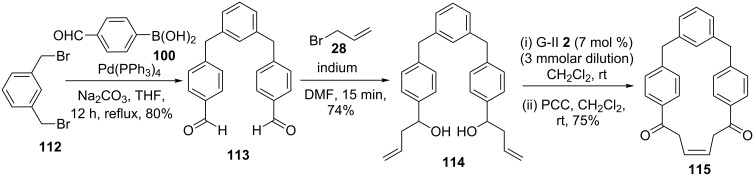
Synthesis of cyclophane **115** via SM coupling and RCM as key steps.

To develop new synthetic strategies to various cyclophanes, we conceived a sequential usage of the SM coupling and RCM as key steps [48–49]. In this context, the required dialdehyde **113** (80%) was prepared via a SM coupling of the dibromo compound **112** with 4-formylphenylboronic acid (**100**). Treatment of dialdehyde **113** with allyl bromide (**28**) in the presence of indium powder furnished the RCM precursor **114**. Under the influence of the G-II catalyst **2** RCM of diolefinic compound **114** was realized. Then, the cyclized product was subjected to the oxidation sequence with pyridinium chlorochromate (PCC) to generate cylophane derivative **115** in 75% yield (Scheme 17).

**Scheme 18:**
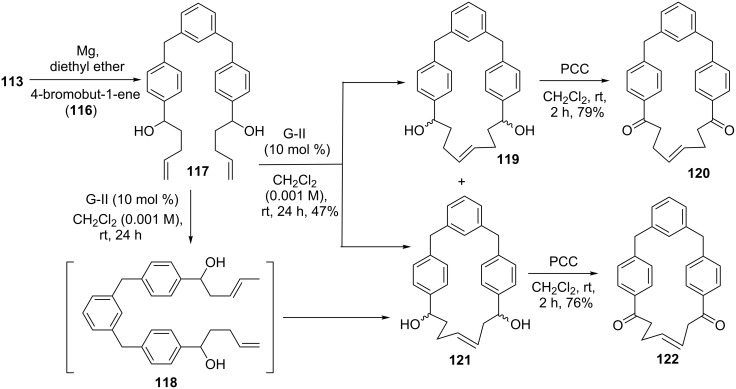
Synthesis of cyclophane **120** and **122** via SM coupling and RCM as key steps.

Similarly, treatment of dialdehyde **113** with a freshly prepared Grignard reagent derived from 4-bromobut-1-ene (**116**) afforded dialkenyl substrate **117**, which was subjected to RCM with the aid of G-II catalyst **2** to produce a mixture of products **119** and **121** in combined 47% yield. It should be noted that the resulting product **121** was obtained through isomerization of the terminal double bond followed by RCM. Later, oxidation of diols **119** and **121** was accomplished with PCC to provide the corresponding diones **120** (79%) and **122** (76%) with *trans* geometry. The stereochemistry was confirmed on the basis of the coupling constant (*J* = 15.0 Hz, ^1^H NMR spectrum) of the olefinic protons (Scheme 18).

**Scheme 19:**
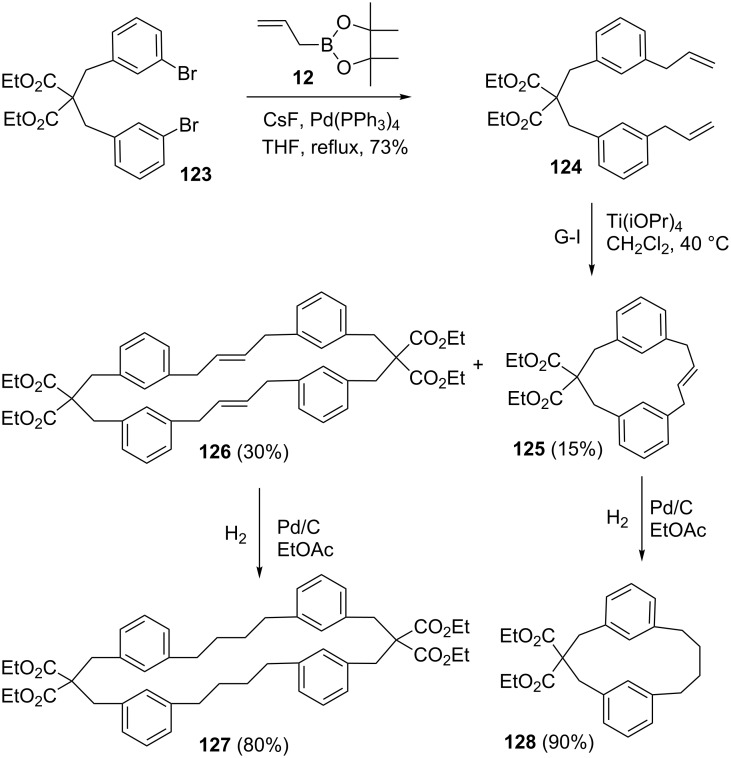
Synthesis of cyclophanes via SM and RCM.

A variety of macrocycles were synthesized through SM cross coupling followed by RCM as key steps [50]. To this end, dibromo compound **123** was subjected to diallylation by using allylboronate ester **12** to form the diallyl derivative **124** (73%). Treatment of compound **124** with G-I catalyst **1** gave unsaturated dimer **126** (30%) and monomer **125** (15%). Subsequently, hydrogenation of compounds **126** and **125** was accomplished with H_2_ under Pd/C catalysis conditions to afford the respective saturated macrocyclic products **127** (80%) and **128** (90%). Since the small ring cyclophane is highly strained, compound **125** was formed as a minor product (Scheme 19).

**Scheme 20:**
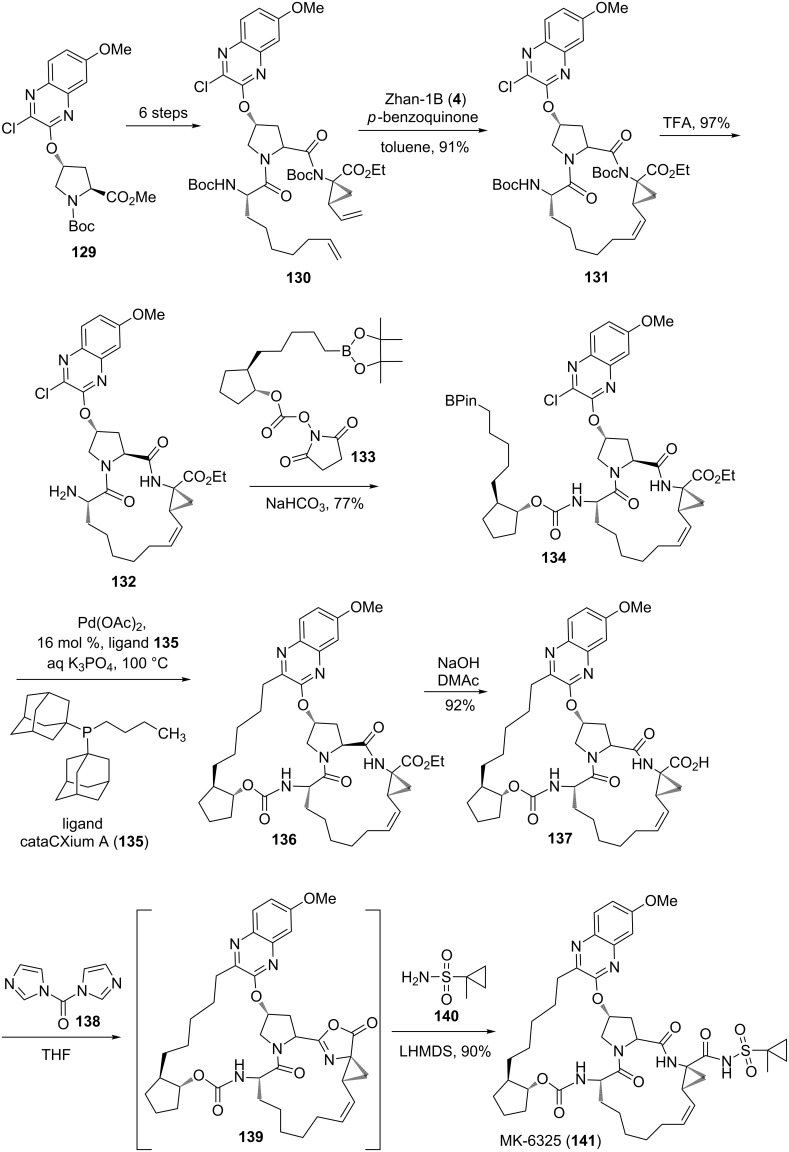
Synthesis of MK-6325 (**141**) via RCM and SM coupling.

Recently, Li et al. [51] disclosed an elegant synthesis of MK-6325 (**141**) through a sequential usage of RCM and SM coupling as key steps. In this respect, the required RCM precursor **130** was derived from **129** by employing a six-step synthesis sequence. Next, the alkene derivative **130** was subjected to RCM under the influence of Zhan-1B catalyst **4** to deliver the cyclized product **131** (91%). Later, TFA-mediated deprotection of cyclized product **131** gave amine **132** (97%). Treatment of chloro derivative **132** with boronate ester **133** provided the SM coupling precursor **134** (77%). Later, an intramolecular SM coupling of Bpin derivative **134** was realized in the presence of a Pd(OAc)_2_ catalyst with the aid of the ligand cataCXium A (**135**) to generate the macrocyclic product **136**. Eventually, synthesis of MK-6325 (**141**) was achieved by adopting saponification followed by amidation (Scheme 20).

## Conclusion

In this review, we have summarized various approaches to a wide range of carbocycles and heterocycles that deals with a strategic utilization of SM coupling and metathesis as key steps. Interestingly, application of these two powerful methods in combination for a C–C bond formation process shorten the synthesis sequence for the assembly of the target molecules and thus enhances the ease of preparation of various functional molecules. These processes are considered as “green” because of atom economy and synthetic brevity [52] involved in these reactions [12,53–54]. Additionally, several methods are available to remove palladium and ruthenium impurities in minor amounts from the reaction mixture. This aspect is also important in the pharmaceutical industry [4,55].

## Biography of the Authors


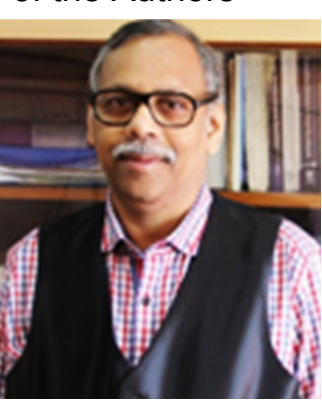


**Sambasivarao Kotha** graduated with M.Sc. degree in Chemistry from the University of Hyderabad and obtained his Ph.D. in Organic Chemistry from the University of Hyderabad in 1985. Later, he moved to UMIST Manchester, UK and the University of Wisconsin, USA as a research associate. Subsequently, he was appointed as a visiting scientist at Cornell University and as a research chemist at Hoechst Celanese Texas prior to joining IIT Bombay in 1994 as an assistant professor. Later, in 2001, he was promoted to Professor. He has published 250 publications in peer-reviewed journals and elected fellow of various academies (FNASc, FASc, FRSC and FNA). He was also associated with editorial advisory boards of several journals. His research interests include: organic synthesis, green chemistry, development of new synthetic methods for unusual amino acids, peptide modifications, cross-coupling reactions, and metathesis. Currently, he occupies the Pramod Chaudhari Chair Professor in Green Chemistry.


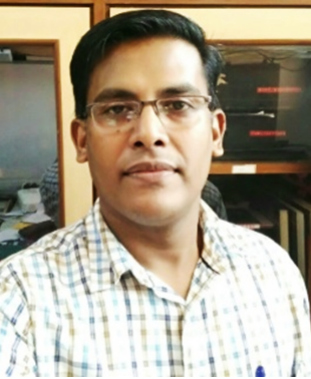


**Milind P. Meshram** was born in Amravati, Maharashtra, India. He obtained his M.Sc. degree in Chemistry from the Amravati University. He joined the Department of Chemistry, IIT Bombay in 2007 and graduated with Ph.D. degree in 2014 (Organic Chemistry) under the supervision of Prof. S. Kotha. Later, he worked with Prof. Dr. Van der Eycken as a Post-Doctoral Fellow at the KU Leuven, Belgium under the EMINTE programme. During post-doctoral work his research work was related to organic synthesis under microwave reaction conditions. Presently, he is Research Associate with Prof. S. Kotha. His research interests include various transition-metal-catalyzed reactions and their applications in organic synthesis.


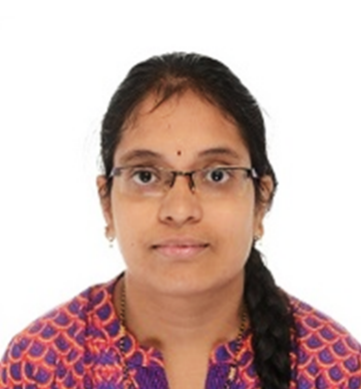


**Chandravathi Chakkapalli** obtained her B.Sc. and M.Sc. in Chemistry from Andhra University, Andhra Pradesh, India. She completed her Ph.D. under the supervision of Dr. J. S. Yadav from IICT, Hyderabad in 2016. Her research interests are in the area of organic synthesis and green chemistry.
